# Causal mechanisms of a scapular stabilization intervention for patients with subacromial pain syndrome: a secondary analysis of a randomized controlled trial

**DOI:** 10.1186/s40945-022-00138-1

**Published:** 2022-06-01

**Authors:** Gisele Harumi Hotta, Rafael Krasic Alaiti, Daniel Cury Ribeiro, Kevin James McQuade, Anamaria Siriani de Oliveira

**Affiliations:** 1 Health Sciences Department, School of Medicine of Ribeirao Preto, University of São Paulo, Ribeirão Preto, SP Brazil; 2 Nucleus of Neuroscience and Behavior and Nucleus of Applied Neuroscience, University of São Paulo, São Paulo, SP Brazil; 3 University of Otago, Centre for Health, Activity, and Rehabilitation Research, Dunedin, New Zealand; 4 Department of Rehabilitation Medicine, School of Medicine, University of Washington, Seattle, USA; 5 Prédio da Fisioterapia e Terapia Ocupacional da Faculdade de Medicina de Ribeirão Preto da Universidade de São Paulo, Avenida Bandeirantes, 3900, Bairro Monte Alegre, Ribeirão Preto, SP 14049-900 Brazil

**Keywords:** Shoulder pain, Mediation analysis, Prognosis analysis, Rehabilitation

## Abstract

**Background:**

Causal mediation analysis is one way to bridge this gap by exploring the causal pathways of a given intervention. The aim of this study was to assess whether scapular motion, position, and periscapular muscle strength are mediators for pain and shoulder disability outcomes following a scapular stabilization intervention for patients with subacromial pain syndrome.

**Methods:**

Sixty patients were randomized into two groups: scapular stabilization or periscapular strengthening exercises. The intervention consisted of three sessions per week for 8 weeks. The primary outcome measures were pain and disability and the following outcome measures were considered as potential mediators: scapular motion, scapular position, periscapular muscle strength, age, duration of symptoms, and side of the complaint. A model-based inference approach with bootstrap simulations was used to estimate the average causal mediation effect, average direct effect, and the average total effect from the data of a randomized clinical trial that evaluated the effect of adding scapular stabilization exercises to a scapulothoracic strengthening program in people with subacromial pain syndrome.

**Results:**

The results demonstrated that none of the putative mediators were influenced by the intervention. However, muscle strength of serratus anterior, upper, middle, and lower trapezius muscles was associated with shoulder disability.

**Conclusion:**

Scapular kinematic and periscapular muscle strength did not mediate the effect of scapular stabilization exercises on shoulder pain or disability scores in subjects with subacromial pain syndrome. Muscle strength of serratus anterior, upper, middle and lower trapezius were associated with shoulder disability scores at 8-weeks follow-up.

**Supplementary Information:**

The online version contains supplementary material available at 10.1186/s40945-022-00138-1.

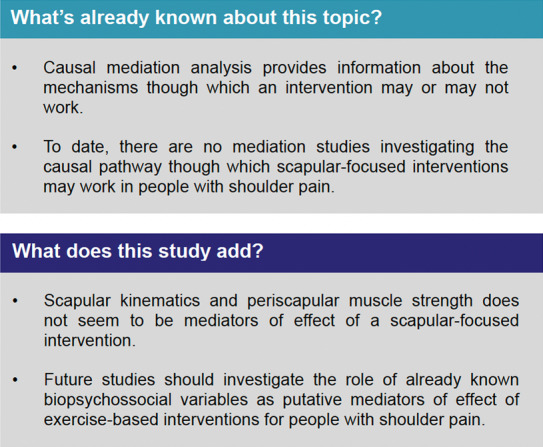



## Background

Subacromial pain syndrome (SAPS) is the most prevalent disorder in the shoulder region, accounting for up to 50–70% of all shoulder complaints in primary care [1]. Exercises are frequently indicated as the first line of treatment for improving pain and disability in people with SAPS, and usually include a combination of scapular stabilization exercises, rotator cuff strengthening and stretching [2, 3]. However, the causal mechanisms associated with exercise-based interventions for shoulder pain complaints are not fully understood.

The scientific literature suggests that an adequate scapular motion may be crucial to shoulder function [4, 5]. Subjects with SAPS present less scapular upward rotation and increased scapular internal rotation [6, 7] and anterior tilt [8] than asymptomatic subjects. Recently, the use of better statistical tools for biomechanical data analysis (i.e. Principal Component Analysis) showed differences in scapular anterior/posterior tilt and forward/backward translation in subjects with SAPS [9]. Despite the lack of high-quality evidence to support a causal association between these biomechanical changes with pain and disability in people with SAPS, it is believed that scapular motion impairments contribute to the development and maintenance of shoulder pain [4], as highlighted by a consensus statement from 2013 [10] and by the Movement System Diagnosis framework [10, 11]. Therefore, interventions for subjects with shoulder pain commonly focus on improving scapular movement [10, 11]. However, although there is some emerging evidence to support scapular-focused exercises for reducing shoulder pain and disability in the short-term [12–14], most of the available studies have a high risk of bias. This is why all available systematic reviews recommended that more trials with better methodologies and larger sample sizes were needed to provide more quality of evidence about the efficacy of scapular stabilization exercises in people with SAPS [15, 16].

Considering the limitations of those studies, we conducted a randomized controlled trial (RCT) to evaluate the incremental effect of scapular stabilization exercises to a scapulothoracic strengthening program in people with SAPS [17]. The results of this study showed that no effects were found when added a scapular stabilization exercise to a scapulothoracic strengthening program for patients with shoulder pain on pain and shoulder disability. These results bring into question the role of scapula on symptoms improvement in patients with SAPS.

Traditional RCTs analyses do not provide insight into the mechanisms through which an intervention works, given that its focus is to identify between-group differences in clinical outcomes [18, 19]. Causal mediation analysis (CMA) is one way to bridge this gap by exploring the causal pathways of a given intervention [18]. Therefore, the main goal of this study was to verify why the intervention did not worked by analyzing the effect of the scapular stabilization exercises on scapular motion, position and muscle strength, as well as to verify the effect of these intermediate variables on the outcomes using CMA.

## Methods

### Participants

The randomized controlled trial involved 60 individuals with SAPS, recruited from March 2016 to June 2017. Patients were referred by an orthopedic physician to physiotherapy due to shoulder pain. We included patients with positive results for 3 out of 5 SAPS tests: Neer, Hawkins-kennedy, painful arc, pain or weakness resistant to external rotation and Jobe [20]. The exclusion criteria were: history of shoulder trauma or surgery; total rotator cuff or biceps brachii tendon rupture (imaging exam or self-report); sports activities involving the upper limbs; individuals with neurological disorders and alterations in cognitive function (e.g., stroke, epilepsy, multiple sclerosis, Parkinson’s disease, and peripheral neuropathy); shoulder pain for primary involvement in the cervical or thoracic region; systemic disease involving the joints (e.g., rheumatoid arthritis); carpal tunnel syndrome; and underwent physiotherapeutic treatment of the shoulder in the last 6 months [17]. A detailed description of the trial was presented by Hotta et al. [17] (clinicaltrials.gov: NCT02695524). A brief overview of the trial is presented below.

### Randomization and interventions

Participants included in the study were randomized into two groups: periscapular strengthening and scapular stabilization exercises. Interventions were carried out for 8 weeks, three times a week, on non-consecutive days. Each session lasted 50 min and individuals were treated separately. Participants assigned to the periscapular strengthening group (PSG) performed only six exercises of periscapular strengthening (upper trapezius, middle trapezius, lower trapezius, and serratus anterior). Participants allocated to the scapular stabilization group (SSG) performed the same six periscapular strengthening exercises applied to PSG, and six scapular stabilization exercises, emphasizing retraction and depression of the scapula, were added to this group [17].

### Assessment time points

Patients characteristics, outcome measures, primary and alternative mediators, and potential confounders were measured at baseline prior to randomization. The putative mediators were measured after 4 weeks of the beginning of the treatment. Outcomes were measured right after the end of the treatment (i.e. 8 weeks).

### Primary outcome measures

Shoulder disability was assessed by The Shoulder Pain and Disability Index (SPADI). The SPADI is valid and reliable (Cronbach Alpha = 0.89) for the assessment of individuals with shoulder disorders (Martins et al., 2010). The score of the questionnaire ranges from 0 to 100 points, and higher scores indicate higher disability [21]. The minimal clinically important difference (MCID) considered for the questionnaire was 10 points [22].

Pain intensity was assessed by the 0–10 numerical pain rating scale [23, 24]. Changes of 15 to 20% from baseline values were considered clinically relevant [25].

### Putative mediators

The primary mediators were scapular motion and position, measured through a digital inclinometer (Lafayette®, Lafayette Instrument Company, Ind., USA) and expressed in degrees. We measured scapular upward/downward rotations and anterior/posterior tilt at rest (scapular position), 90° and 180° of arm elevation (scapular motion). Scapular upward/downward rotations and anterior/posterior tilt measurements presented intra-rater reliability ranging from good to excellent, with standard error of measurement ranging 2 to 2.8 degrees [26, 27] and criterion validity ranging from good to excellent [26, 28]. Other mediators included muscle strength of serratus anterior, upper, middle, and lower trapezius [29]. The evaluation of the isometric strength was performed using a portable dynamometer that has excellent reliability [26, 27] (Lafayette®, Lafayette Instrument Company, Ind., USA).

### Potential confounders

**Fig. 1:**
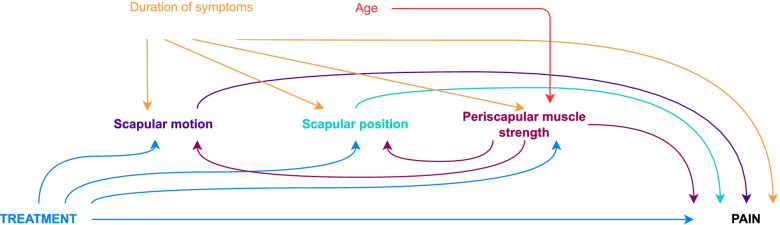
Directed Acyclic Graph (DAG) of the hypothesized causal pathway of scapular stabilization exercises effect on pain. The scapular motion, position and periscapular muscle strength are putative mediators, while the pain duration and age are confounders of the mediator-outcome path. The periscapular muscle strength was also considered as a confounder of the relation between scapular motion and position and the outcome

**Fig. 2:**
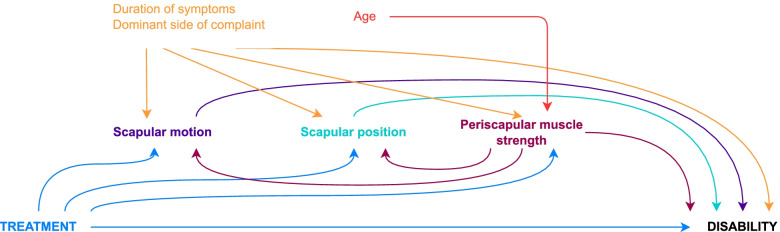
Directed Acyclic Graph (DAG) of the hypothesized causal pathway of scapular stabilization exercises effect on shoulder disability. The scapular motion, position and periscapular muscle strength are putative mediators, while the pain duration, age and side of complaint are confounders of the mediator-outcome path. The periscapular muscle strength was also considered as a confounder of the relation between scapular motion and position and the outcome

We assumed that both the intervention-mediator and intervention-outcome paths were not confounded due to randomization. Given the mediator cannot be randomized, we assumed that the mediator-outcome path might be confounded by pain duration, age, sex and baseline measures of mediators and outcomes. Therefore, we controlled the analyses in the outcome regression models for pain duration, age, sex and baseline measures of mediators and outcomes. Besides that, the outcome regression models for pain and disability, with scapular position and motion as mediators, were also controlled for muscle strength and all outcome models were controlled for the dominant side of complaint. The hypothesized causal pathways are presented in Figs. 1 and 2.

### Sample size

The sample size for the main trial was estimated to identify clinically meaningful between-group differences in shoulder function, considering a minimal clinically important difference of 10 points in the global SPADI score, with alpha set at 0.05, power 80%, and a 20% sample loss. The minimum sample size for the original trial was 30 on each intervention arm. The trial had no drop-outs, with 60 participants (30 each group) completing the study.

We conducted a post hoc power calculation as suggest by the literature [30, 31] using the estimator for joint indirect effect developed by Vittinghoff and Neilands [32]. We performed two sample size estimations: (1) one assuming a large treatment-mediator and mediator-outcome effect (r = 0.65); (2) another assuming a moderate treatment-mediator and mediator-outcome effect (r = 0.3). The remaining variables were kept the same for both analysis, and were as follows: absence of exposure-mediator confounding (i.e. error term correlation coefficient = 0.0), given the design of the study (i.e. clinical trial); a moderate confounding for the mediator-outcome (r = 0.3) as suggested by Vittinghoff and Neilands [32]; with power set at 0.8. The coefficients were standardized.

### Data analysis

**Fig. 3:**
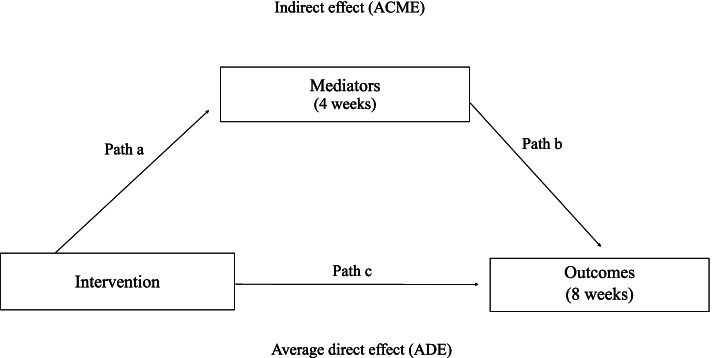
The path a was the effect intervention on the mediator. The path b was the effect of the mediator on the outcome. The Indirect Effect (ACME) was the effect of the intervention on the outcome through the putative mediator. The direct effect (ADE) is the remaining effect of the intervention, mediated through unmeasured mediators, on pain and shoulder disability

Analyses were performed in R (The R Foundation for Statistical Computing). The causal mediation analysis was performed using the *mediation* package [33]. A model-based inference approach was used to estimate the average causal mediation effect (ACME), average direct effect (ADE) and the average total effect (Fig. 3) [33, 34]. Two regression models were created: the mediator model and the outcome model. As there was no total effect, we decided to conduct several univariate mediation models to verify where the causal pathway break down. The mediator model was constructed with treatment allocation as the independent variable and the putative mediator as the dependent variable. The outcome model was constructed with the treatment allocation and the putative mediator as independent variables and the outcome as independent variable. The outcome models were adjusted for potential confounders. Continuous mediators and outcomes that were normally distributed were modelled using linear models (*lm*). However, if the data was skewed or the assumptions of the linear model were violated, mediators and outcomes were modelled using robust linear models^22^ or generalized linear models (*glm*) with respective family and link function [35].

The *mediate* function was used to estimate the value of the mediator and outcome. The simulated potential values of the mediator and outcome was used to compute the ACME, ADE and average total effect. We used 1000 bootstrap simulations to generate 95% confidence intervals (95% CI) if linear assumptions of mediator and/or outcome models were not violated. Non-parametric bootstrap simulations were used if the linear assumptions of the mediator and/or outcome models were violated.

We performed sensitivity analysis for unmeasured confounding to assess how a hypothetical level of unmeasured confounding would impact on ACME. We used the *medsens* function to explore the level of confounding due to unknown confounders from the mediator ant outcome models. The level of confounding [ρ (rho)] is represented by the correlation between the error terms from the mediator and outcome models. The level of confounding ranged from − 1 to 1. A ρ = 0 suggests no correlation between error terms and can be interpreted as the absence of unmeasured confounding [34].

## Results

The intervention had no additional effect on pain intensity and shoulder disability. The causal mediation analysis demonstrated that the intervention did not influence the primary mediators (ie. scapular motion and position) or the alternative mediators (ie. serratus anterior, upper, middle, and lower trapezius strength). The alternative mediators serratus anterior, upper, middle and lower trapezius strength were associated with shoulder disability.

The full description of the causal mediation analysis is described in Table 1.

**Table 1:** Effect decomposition for each single-mediator model

Analysis	Intervention-mediator effect (Path a)	Mediator-outcome effect (Path b)	ATE	ADE	ACME	Proportion Mediated (%)
**Pain**						
**Scapular Position**						
SUR 0**°**	0.01 (−1.9 to 2.9)	0.23 (0.02 to 0.45)	−0.34 (− 1.89 to 1.24)	− 0.36 (− 2 to 1.29)	0.02 (− 0.32 to 0.35)	−0.05 (− 1.89 to 2)
SAT 0**°**	0.07 (− 2.4 to 2.6)	0.01 (− 0.14 to 0.18)	− 0.31 (− 1.85 to 1.44)	−0.32 (− 1.9 to 1.4)	0.006 (− 0.26 to 0.37)	−0.01 (− 1.23 to 1.96)
**Scapular Motion**						
SUR 90**°**	0.5 (− 3.2 to 3.3)	−0.06 (− 0.18 to 0.05)	−0.01 (− 0.3 to 0.12)	−0.24 (− 1.82 to 1.48)	−0.26 (− 1.84 to 1.49)	0.06 (−1.02 to 1.52)
SUR 180**°**	− 0.04 (− 4.8 to 4.7)	−0.19 (− 0.28 to 0.1)	−0.63 (− 2.4 to 1.9)	−0.7 (− 2.26 to 1.18)	0.05 (− 0.32 to 0.30)	−0.07 (− 0.07 to 2.32)
SAT 90**°**	− 0.16 (− 2.51 to 2.47)	−0.13 (− 0.3 to 0.03)	−0.25 (− 1.97 to 1.43)	−0.26 (− 2 to 1.93)	0.01 (− 0.2 to 0.33)	0.004 (−1.5 to 2)
SAT 180**°**	0.09 (− 4.2 to 4.4)	0.44 (− 0.35 to 0.53)	−0.23 (− 2 to 1.47)	−0.46 (− 2 to 1)	0.23 (− 0.17 to 1)	−0.99 (− 4.3 to 3.91)
**Muscle Strength**						
SA	0.12 (− 1.5 to 1.6)	−0.51 (− 0.73 to 0.02)	−0.3 (− 1.8 to 1)	−0.08 (− 1.57 to 1.24)	−0.3 (− 1.4 to 1.4)	0.3 (− 3 to 4)
UT	0.09 (−1.5 to 1.7)	− 0.31 (− 0.55 to 0.06)	−0.25 (− 0.2 to 0.2)	−0.19 (− 0.3 to 0.7)	−0.9 (− 0.1 to 0.09)	0.10 (− 0.41 to 0.9)
MT	−0.03 (− 0.68 to 0.7)	−0.19 (− 0.28 to 0.1)	−0.05 (− 1.65 to 1.4)	−0.6(− 1.8 to 1.1)	0.36 (− 0.2 to 1.2)	−0.10 (− 9.68 to 6.8)
LT	− 0.6 (− 0.69 to 0.8)	−0.19 (− 0.28 to 0.1)	−0.27 (− 1.79 to 1.2)	−0.3 (− 1.89 to 0.89)	0.2 (− 0.27 to 0.71)	−0.63 (− 3.88 to 4.59)
**Shoulder Disability**						
**Scapular Position**						
SUR 0**°**	0.01 (−1.9 to 2)	−0.04 (− 1.6 to 1.5)	− 0.3 (− 2.2 to 3.3)	−0.42 (− 2.27 to 1.32)	0.06 (− 0.35 to 0.64)	0.03 (−1.33 to 1.19)
SAT 0**°**	0.07 (− 2.4 to 2.6)	0.19 (−1 to 1)	5 (− 6 to 16.2)	3 (− 6.16 to 14.9)	0.8 (− 1.6 to 5.3)	0.04 (− 1.55 to 1.97)
**Scapular Motion**						
SUR 90**°**	0.49 (−3 to 3.38)	0.19 (− 0.73 to 1.13)	6 (−6 to 10)	5 (− 6 to 14.36)	0.6 (− 3.4 to 4.59)	0.01 (− 1.6 to 2.41)
SUR 180**°**	−0.04 (− 4.8 to 4.7)	− 0.14 (− 0.84 to 0.55)	4.6 (− 7 to 14)	4 (−8 to 13)	0.6 (− 1.88 to 5.3)	0.05 (− 1.55 to 1.97)
SAT 90**°**	−0.01 (− 2.55 to 2.24)	0.31 (− 0.17 to 2.47)	7 (− 5.5 to 16)	6.9 (− 5.5 to 16)	0.1 (− 3.3 to 2.73)	0.02 (− 1.97 to 2)
SAT 180**°**	0.09 (− 4.2 to 4.4)	0.25 (− 0.44 to 0.96)	6 (− 5 to 16.3)	4 (−6 to 14)	1.08 (− 5.82 to 6.44)	0.07 (− 1.2 to 2)
**Muscle Strength**						
SA	0.12 (− 1.56 to 1.8)	− 0.64 (− 2.2 to − 0.97)***	1 (− 9.44 to 13)	0.15 (− 4 to 17)	− 0.05 (− 2 to 1.67)	− 0.05 (− 1 to 1.2)
UT	0.09 (−1.5 to 1.6)	− 0.44 (− 5 to − 1.3)**	2.86 (− 8 to 14)	5.1 (− 5.15 to 17.3)	−2.3 (− 7 to 2)	− 0.8 (− 9.78 to 12)
MT	− 0.03 (− 0.68 to 0.75)	−0.42 (− 4.9 to − 0.2)*	0.25 (−5.79 to 6)	1 (− 10 to 12)	−0.8 (− 4 to 2)	− 3.2 (− 3.3 to 3)
LT	− 0.06 (− 0.69 to 0.8)	−0.3 (− 4 to − 1)**	−0.2 (− 5.79 to 6)	1 (− 10.8 to 13)	− 1.1 (− 5 to 1)	0.5 (− 3.3 to 3)

*ATE* Average total effect, *ADE* Average direct effect, *ACME* Average causal mediation effect, *SUR* Scapular upward rotation, *SAT* Scapular anterior tilt, *SA* Serratus anterior, *UTS* Upper trapezius, *MT* Middle trapezius, *LT* Lower trapezius

Standardized coefficients with their 95% confidence intervals unless otherwise stated

*p < 0.05; **p < 0.01; ***p < 0.001

The sensitivity analysis showed that our estimated causal mediation effects were stable across all possible levels of residual confounding for the pain models. The disability models were relatively unstable for some levels of residual confounding. The sensitivity analysis plots for each model are reported in Figures S1 and S2 in the Supplementary file.

The analyses of post hoc power calculation suggested that assuming large treatment-mediator and mediator-outcome effects, a minimum of 67 participants were required. Assuming moderate treatment-mediator and mediator-outcome effects, a minimum of 342 participants were required. Those analyses showed that our study is underpowered for detecting a large and moderate mediating effect. Considering that, the coefficients presented in Table 1 were standardized.

## Discussion

The main purpose of this secondary analysis was to assess the mechanism of action of a scapular intervention program for patients with subacromial pain syndrome, considering scapular motion, position and periscapular muscle strength as possible mediators in people with SAPS. The results of the causal mediation analysis demonstrated that scapular motion, position and periscapular muscle strength were not influenced by the intervention (i.e., path a), while muscle strength of SA, UT, MT and LT was associated with shoulder disability (i.e., path b).

Our findings challenge the assumption that scapular-focused interventions may lead to better clinical outcomes by altering scapular movement pattern, position and muscle strength [13, 25, 36]. This assumption is partially supported by previous clinical trials that reported conflicting evidence that interventions targeting scapular movement may lead to better clinical outcomes [13, 14]. However, these previous clinical trials have high risk of bias due to sample size, lack of allocation concealment, heterogeneity in term of outcome measurement and lack of intention-to-treat analysis [13, 14]. Our findings suggest the path between intervention and mediators (i.e., scapular movement pattern, position, or muscle strength) were not significant. The causal paths between muscle strength and disability scores were unstable and may be biased by unmeasured mediator-outcome confounding.

Due to the multifactorial nature of SAPS and the uncertain relationship between pain and shoulder disability with movement and posture, some methodology for measuring scapular motion and position have been studied in the literature [5]. A recent cross-section study reported a fair association between improvement in shoulder function and improvement in scapular dyskinesis, and no association between pain and scapular dyskinesis [37]. Therefore, future studies should explore the causal effects between improvement in shoulder function and scapular dyskinesis, since we do not evaluate the presence of this condition in our participants.

The use of RCT design allows controlling for intervention-outcome confounding, but not for mediator-outcome confounding. The results of the Hotta et al. [17] clinical trial demonstrated that there were no differences between the group that received additional scapular stabilization exercises with a focus on scapular retraction and scapular depression movements when compared to the muscle strengthening control group. Therefore, despite the effect of the intervention on the mediators was not significant, the causal mechanism of scapular orientation and muscle strength with pain and disability remains uncertain.

Some authors have questioned the theoretical concept of scapular stabilization and the function of a “stable base” for upper limb movement [38]. The “scapular stability” paradigm is considered flawed due to limitations with regards to the definition of concept of joint stability and the knowledge that shoulder movement is complex and may be better represented through a dynamic systems perspective [38, 39]. Considering that there is a greater number of muscles than degrees of freedom that involves the shoulder girdle and upper limb joints, to complete a task, from the point of view of dynamic systems theory, the central nervous system could use a variety of segmental coordination patterns, within an optimal range of variability [38, 39]. Thus, the prescription of exercises aimed to stabilize the scapula and influence its movement pattern, in patients with scapular dyskinesis, for instance, are being made in a completely arbitrary way, since the role of scapular motion and position on shoulder pain and disability is still not fully understood.

The motor control intervention proposed in our clinical trial [17] was based on scapular retraction and depression movements, one possible explanation for the absence of intervention-mediators effect upon scapular position is that this intervention did not offered adequate motor stimulus to induce changes in motor behavior. Experimental paradigms have evaluated movement variability, systematic movement error and motor action planning and defined that kinematic accuracy and learning have limited generalization and are encoded in extrinsic coordinates [40]. Internal learning models transform biomechanical properties (i.e. strain, stiffness) in motion and the ability to anticipate dynamic effects, such as torque [40]. Current literature suggests that motor learning and the consolidation of kinematics and dynamics occur independently [40] and that the generalization of motor learning is influenced not only by prediction errors, but also by the history of implicitly remembered contexts in which the training occurred [41]. Those principles from dynamic systems suggest that isolated scapular stabilization exercises are unlikely to change scapular movement pattern. A better approach would be to improve motor behavior by exposing patients to functional movements of the upper limb [38].

### Limitations

This study had some limitations. Our main goal was to better understand the causal pathways through which a scapular stabilization program may have affected pain and disability scores. Therefore, one challenge of our study was to measure scapular movement and this is a common issue in the field. We measured two scapular rotations (upward-downward rotation, and anterior-posterior tilt) at different glenohumeral positions. Given the challenges in measuring scapular movement, we had to conduct several univariate causal mediation analyses when assessing whether scapular movement mediated the effects of the treatment. The post hoc power calculation showed that we are underpowered for the mediation analysis, considering a large treatment effect. That increases the odds of Type 2 error. Nevertheless, when a causal question is important, it is preferable to have multiple studies with imprecise estimates than having no study at all [30]. Considering the limitations, this study can be a guide for the development of future studies with power in this area.

## Conclusion

Scapular motion, position and periscapular muscle strength did not mediate the effect of scapular stabilization exercises on shoulder pain and disability in subjects with subacromial pain syndrome. Muscle strength of serratus anterior, upper, middle, and lower trapezius mediated changes in shoulder disability, but these estimates were unstable due to possible unmeasured mediator-outcome confounding.

## Supplementary Information

**Additional file 1: Figure S1.** Sensitivity analysis plots for each single mediator model for pain. The correlation between the error terms in the mediator and outcome regression models (ρ) is plotted against the average causal mediation effect (ACME). The estimated ACME (assuming sequential ignorability) is the dashed line and the 95% confidence intervals are represented by the shaded regions. **Figure S2.** Sensitivity analysis plots for each single mediator model for shoulder function. The correlation between the error terms in the mediator and outcome regression models (ρ) is plotted against the average causal mediation effect (ACME). The estimated ACME (assuming sequential ignorability) is the dashed line and the 95% confidence intervals are represented by the shaded regions.

## Data Availability

The datasets generated during and/or analysed during the current study are available from the corresponding author on reasonable request.

## Abbreviations

ACME: Average causal mediation effect
ADE: Average direct effect
CMA: Causal mediation analysis
DAG: Directed Acyclic Graph
LT: Lower trapezius
MT: Middle trapezius
PSG: Periscapular group
RCT: Randomized controlled trial
SAPS: Subacromial pain syndrome
SSG: Scapular Stabilization group
SPADI: Shoulder pain and disability index
SA: Serratus anterior
UT: Upper trapezius

## References

[1] Chipchase LS, O’Connor DA, Costi JJ, Krishnan J (2000). Shoulder impingement syndrome : preoperative health status. J Shoulder Elb Surg.

[2] Pieters L, Lewis J, Kuppens K, Jochems J, Bruijstens T, Joossens L (2019). An update of systematic reviews examining the effectiveness of conservative physiotherapy interventions for subacromial shoulder pain. J Orthop Sport Phys Ther.

[3] Reijneveld EAE, Noten S, Michener LA, Cools A, Struyf F (2017). Clinical outcomes of a scapular-focused treatment in patients with subacromial pain syndrome: a systematic review. Br J Sports Med.

[4] Takeno K, Glaviano NR, Norte GE, Ingersoll CD (2019). Therapeutic interventions for scapular kinematics and disability in patients with subacromial impingement: a systematic review. J Athl Train.

[5] Ratcliffe E, Pickering S, McLean S, Lewis J (2014). Is there a relationship between subacromial impingement syndrome and scapular orientation? A systematic review. Br J Sports Med..

[6] Keshavarz R, Bashardoust Tajali S, Mir SM, Ashrafi H (2017). The role of scapular kinematics in patients with different shoulder musculoskeletal disorders: a systematic review approach. J Bodyw Mov Ther.

[7] Timmons MK, Thigpen CA, Seitz AL, Karduna AR, Arnold BL, Michener LA. Scapular kinematics and subacromial impingement syndrome: a Meta-analysis. J Sport Rehabil. 2012:354–70 Available from: http://www.ncbi.nlm.nih.gov/pubmed/22388171. 10.1123/jsr.21.4.354 22388171

[8] Lefèvre-Colau MM, Nguyen C, Palazzo C, Srour F, Paris G, Vuillemin V (2018). Kinematic patterns in normal and degenerative shoulders. Part II: review of 3-D scapular kinematic patterns in patients with shoulder pain, and clinical implications. Ann Phys Rehabil Med.

[9] Rossi DM, Resende RA, Hotta GH, da Fonseca ST, de Oliveira AS (2020). Altered scapular time-series in individuals with subacromial pain syndrome. J Appl Biomech Appl Biomech.

[10] Ben KW, Ludewig PM, Mcclure PW, Michener LA, Bak K, Sciascia AD (2013). Clinical implications of scapular dyskinesis in shoulder injury: the 2013 consensus statement from the ‘scapular summit’.

[11] Ludewig PM, Kamonseki DH, Staker JL, Lawrence RL, Camargo PR, Braman JP. Changing our diagnostic paradigm: movement system diagnostic classification. Int J Sports Phys Ther. 2017;12:884–93. Available from: https://pubmed.ncbi.nlm.nih.gov/29158950/. PMC5675364 29158950

[12] Ravichandran H, Janakiraman B, Gelaw AY, Fisseha B, Sundaram S (2020). Effect of scapular stabilization exercise program in patients with subacromial impingement syndrome : a systematic review.

[13] Bury J, West M, Chamorro-Moriana G, Littlewood C (2016). Effectiveness of scapula-focused approaches in patients with rotator cuff related shoulder pain: a systematic review and meta-analysis. Man Ther.

[14] Saito H, Harrold ME, Cavalheri V, McKenna L. Scapular focused interventions to improve shoulder pain and function in adults with subacromial pain: a systematic review and meta-analysis. Physiother Theory Pract. 2018;3985:1–18. Available from: https://pubmed.ncbi.nlm.nih.gov/29351510/. 10.1080/09593985.2018.1423656 29351510

[15] Shah M, Sutaria J, Khant A (2014). Effectiveness of Scapular Stability Exercises in the Patient With the Shoulder Impingement Syndrome. Indian J Phys Ther.

[16] Başkurt Z, Başkurt F, Gelecek N, Özkan MH (2011). The effectiveness of scapular stabilization exercise in the patients with subacromial impingement syndrome. J Back Musculoskelet Rehabil.

[17] Hotta GH, de Assiss Couto AG, Cools AM, KJ MQ, de Oliveira AS (2020). Effects of adding scapular stabilization exercises to a periscapular strengthening exercise program in patients with subacromial pain syndrome : a randomized controlled trial. Musculoskelet Sci Pract.

[18] Zhang Z, Zheng C, Kim C, Van PS, Lin S, Lan P (2016). Causal mediation analysis in the context of clinical research. Ann Transl Med.

[19] Lee H, Lamb SE (2017). Advancing physical therapist interventions by investigating causal mechanisms. Phys Ther.

[20] Michener LA, Walsworth MK, Doukas WC, Murphy KP (2009). Reliability and diagnostic accuracy of 5 physical examination tests and combination of tests for subacromial impingement. Arch Phys Med Rehabil.

[21] Martins J, Napoles BV, Hoffman CB, Oliveira AS (2010). The Brazilian version of shoulder pain and disability index: translation, cultural adaptation and reliability. Brazilian J Phys Ther.

[22] Roy JS, Macdermid JC, Woodhouse LJ (2009). Measuring shoulder function: a systematic review of four questionnaires. Arthritis Care Res.

[23] Farrar JT, Young JP, LaMoreaux L, Werth JL, Poole RM (2001). Clinical importance of changes in chronic pain intensity measured on an 11-point numerical pain rating scale. Pain..

[24] Turk DC, Dworkin RH, Allen RR, Bellamy N, Brandenburg N, Carr DB (2003). Core outcome domains for chronic pain clinical trials: IMMPACT recommendations. Pain.

[25] Dworkin RH, Turk DC, McDermott MP, Peirce-Sandner S, Burke LB, Cowan P (2009). Interpreting the clinical importance of group differences in chronic pain clinical trials: IMMPACT recommendations. Pain.

[26] Johnson MP, McClure PW, Karduna AR (2001). New method to assess scapular upward rotation in subjects with shoulder pathology. J Orthop Sports Phys Ther.

[27] Laudner KG, Stanek JM, Meister K (2007). Differences in scapular upward rotation between baseball pitchers and position players. Am J Sports Med.

[28] Scibek JS, Carcia CR (2014). Validation of a new method for assessing scapular anterior-posterior tilt. Int J Sports Phys Ther.

[29] Cools AM, Johansson FR, Cambier DC, Vande VA, Palmans T, Witvrouw EE (2010). Descriptive profile of scapulothoracic position, strength and flexibility variables in adolescent elite tennis players. Br J Sports Med.

[30] VanderWeele TJ (2020). Invited commentary: frontiers of power assessment in mediation analysis. Am J Epidemiol.

[31] Fritz MS, MacKinnon DP (2007). Required sample size to detect the mediated effect. Psychol Sci.

[32] Vittinghoff E, Neilands TB (2015). Sample size for joint testing of indirect effects. Prev Sci.

[33] Tingley D, Yamamoto T, Hirose K, Keele L, Imai K. Mediation: R Package for Causal Mediation Analysis. J Stat Softw. 2014;59(5):1–38. 10.18637/jss.v059.i05.

[34] Imai K, Keele L, Tingley D, Yamamoto T (2011). Unpacking the black box of causality: learning about causal mechanisms from experimental and observational studies. Am Polit Sci Rev.

[35] Ng V, Cribbie RA (2019). Using the gamma generalized linear model for modeling continuous, skewed and heteroscedastic outcomes in psychology. Quant Methods Progr Dep.

[36] Steuri R, Sattelmayer M, Elsig S, Kolly C, Tal A, Taeymans J (2017). Effectiveness of conservative interventions including exercise, manual therapy and medical management in adults with shoulder impingement: a systematic review and meta-analysis of RCTs. Br J Sports Med.

[37] Jafarian Tangrood Z, Sole G, Ribeiro DC (2020). Is there an association between changes in pain or function with changes in scapular dyskinesis: a prospective cohort study. Musculoskelet Sci Pract.

[38] McQuade KJ, Borstad J, de Oliveira AS (2016). Critical and theoretical perspective on scapular stabilization: what does it really mean, and are we on the right track?. Phys Ther.

[39] Sherman A (2011). Dynamical systems theory in physiology. J Gen Physiol.

[40] Krakauer JW, Ghilardi M, Ghez C (1999). Independent learning of internal models for kinematic and dynamic control of reaching. Nat Neurosci.

[41] Krakauer JW, Mazzoni P, Ghazizadeh A, Ravindran R, Shadmehr R (2006). Generalization of Motor Learning Depends on the History of Prior Action. PLoS Biol.

