# Supplementary material for: Causal mechanisms of a scapular stabilization intervention for patients with subacromial pain syndrome: a secondary analysis of a randomized controlled trial
Source: Arch Physiother. 2022 Jun 1;12:13. doi: 10.1186/s40945-022-00138-1 (PMC9158354; doi:10.1186/s40945-022-00138-1)

**Supplemental Material**

**Figure S1.** Sensitivity analysis plots for each single mediator model for pain. The correlation between the error terms in the mediator and outcome regression models (ρ) is plotted against the average causal mediation effect (ACME). The estimated ACME (assuming sequential ignorability) is the dashed line and the 95% confidence intervals are represented by the shaded regions.

**
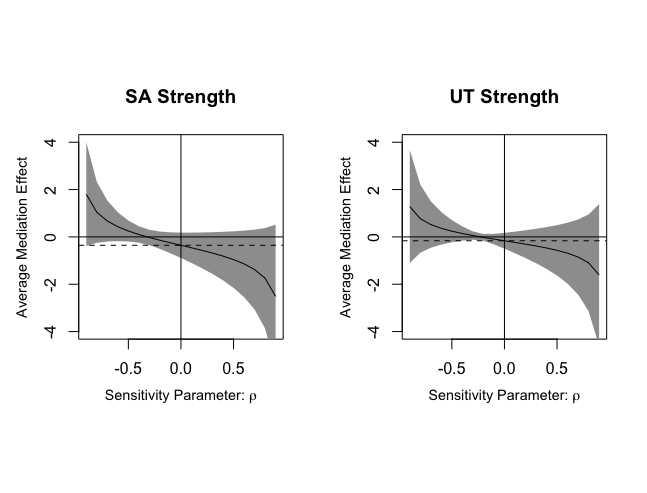

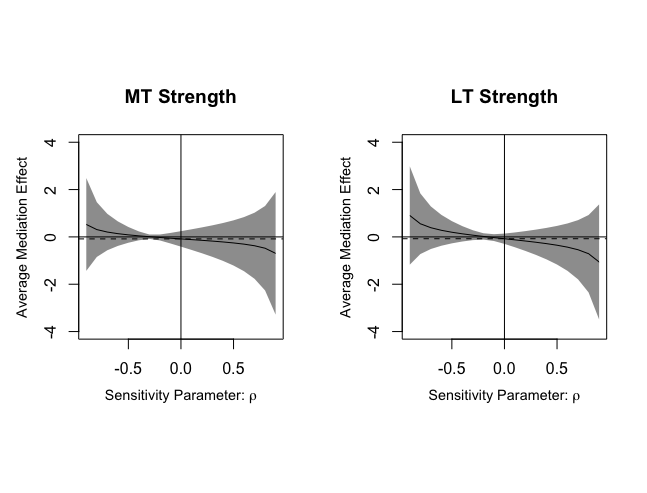

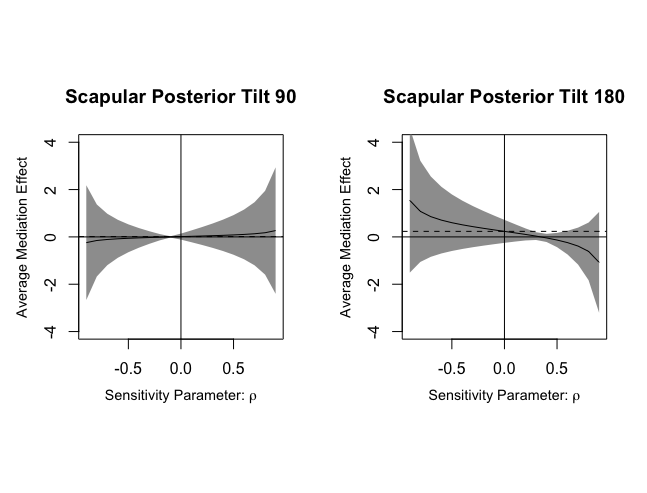

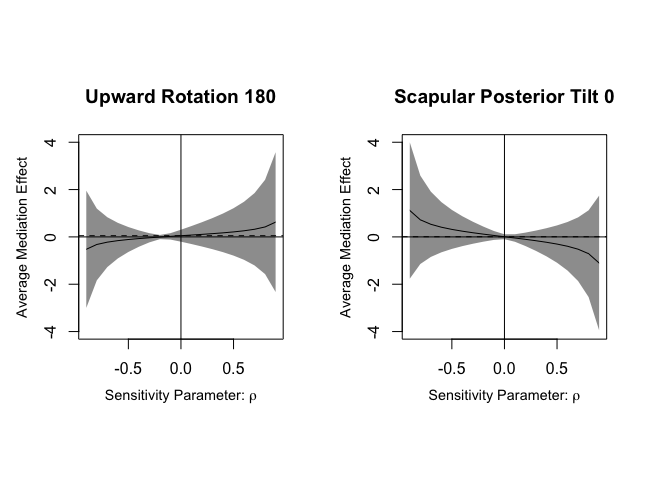
**
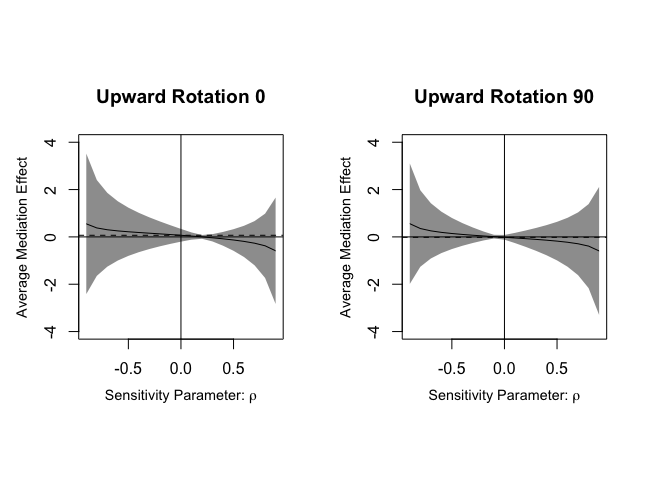


**Figure S2.** Sensitivity analysis plots for each single mediator model for shoulder function. The correlation between the error terms in the mediator and outcome regression models (ρ) is plotted against the average causal mediation effect (ACME). The estimated ACME (assuming sequential ignorability) is the dashed line and the 95% confidence intervals are represented by the shaded regions.


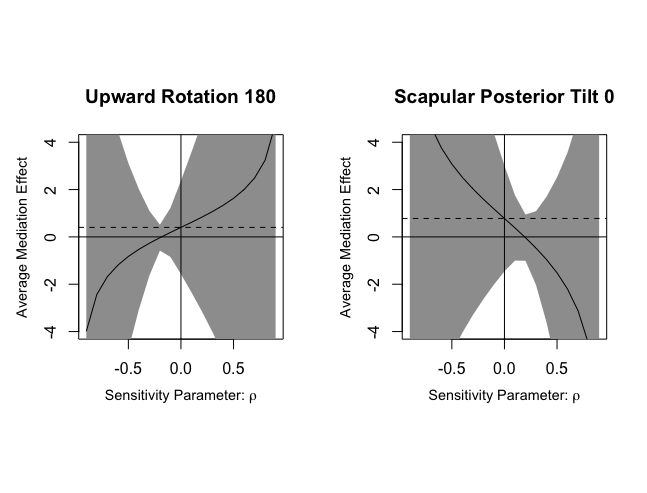

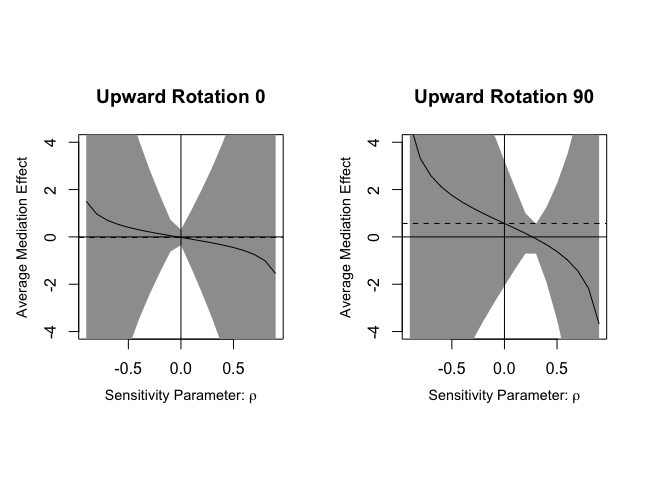


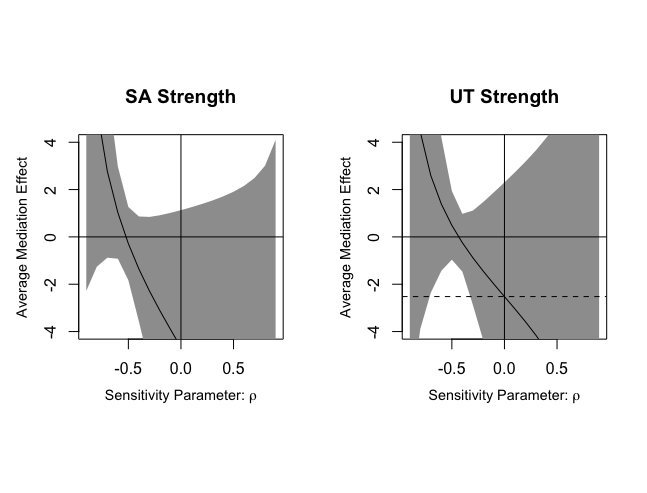

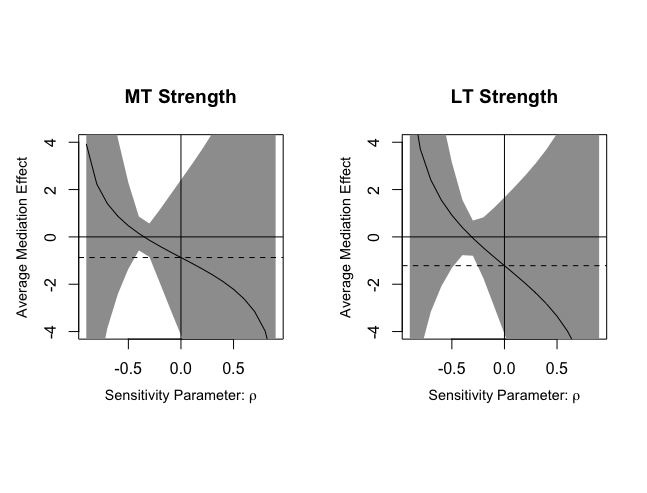

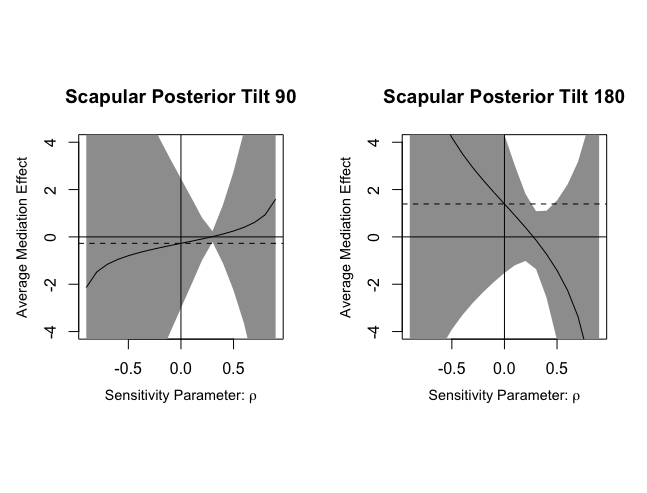

Supplement: Supplementary file 1 — Additional file 1: Figure S1. Sensitivity analysis plots for each single mediator model for pain. The correlation between the error terms in the mediator and outcome regression models (ρ) is plotted against the average causal mediation effect (ACME). The estimated ACME (assuming sequential ignorability) is the dashed line and the 95% confidence intervals are represented by the shaded regions. Figure S2. Sensitivity analysis plots for each single mediator model for shoulder function. The correlation between the error terms in the mediator and outcome regression models (ρ) is plotted against the average causal mediation effect (ACME). The estimated ACME (assuming sequential ignorability) is the dashed line and the 95% confidence intervals are represented by the shaded regions. [file 40945_2022_138_MOESM1_ESM.docx]
